# Primary phonological planning units in spoken word production are language-specific: Evidence from an ERP study

**DOI:** 10.1038/s41598-017-06186-z

**Published:** 2017-07-19

**Authors:** Jie Wang, Andus Wing-Kuen Wong, Suiping Wang, Hsuan-Chih Chen

**Affiliations:** 1 Department of Psychology, The Chinese University of Hong Kong, Hong Kong S.A.R., China; 2 Nam Shan Psychology Laboratory, Department of Applied Social Sciences, City University of Hong Kong, Hong Kong S.A.R., China; 3 Department of Psychology, South China Normal University, Guangzhou, 510631 China

## Abstract

It is widely acknowledged in Germanic languages that segments are the primary planning units at the phonological encoding stage of spoken word production. Mixed results, however, have been found in Chinese, and it is still unclear what roles syllables and segments play in planning Chinese spoken word production. In the current study, participants were asked to first prepare and later produce disyllabic Mandarin words upon picture prompts and a response cue while electroencephalogram (EEG) signals were recorded. Each two consecutive pictures implicitly formed a pair of prime and target, whose names shared the same word-initial atonal syllable or the same word-initial segments, or were unrelated in the control conditions. Only syllable repetition induced significant effects on event-related brain potentials (ERPs) after target onset: a widely distributed positivity in the 200- to 400-ms interval and an anterior positivity in the 400- to 600-ms interval. We interpret these to reflect syllable-size representations at the phonological encoding and phonetic encoding stages. Our results provide the first electrophysiological evidence for the distinct role of syllables in producing Mandarin spoken words, supporting a language specificity hypothesis about the primary phonological units in spoken word production.

## Introduction

As the most distinguished ability of human beings, language cognition has been of great interest to many researchers for a long time. The diversity of human languages raises a critical question: what aspects of language processing are universal or language-specific? A related issue which has gained increasing attention and has been studied cross-linguistically concerns the process of phonological encoding in spoken word production, namely how the phonological form of the target word is constructed prior to motor program preparation^1–3^.

A typical finding from cross-linguistic comparisons on the target issue is that manipulation on a single segment robustly affects the naming responses of speakers of Germanic languages such as Dutch and English^4–8^, but seldom so for Chinese speakers^9–18^. For example, using an implicit priming paradigm, Meyer^6^ found that in a Dutch word generation task the participants responded faster in a homogeneous context where the same onset segment was shared among all the response words (e.g. ***h*** *ut*, ***h*** *eks*, ***h*** *iel*—hut, witch, heel) relative to a heterogeneous context. However, such a facilitation effect of sharing onset failed to be replicated in Chinese^9–11, 16, 17^. Instead, researchers have found robust effects of sharing atonal syllables in Chinese spoken word production^9–12, 15–20^. To account for this discrepancy, O’Seaghdha and colleagues^9^ proposed that the primary phonological units below the word level (called the proximate units, e.g., segments in English, atonal syllables in Chinese) are language-specific and that the above behavioural effects originate from participants’ intentional preparation of these proximate units. They claimed that “there is a requisite step of syllable encoding” prior to subordinate segmental specification in Chinese spoken word production, resulting in the absence of the onset effect.

However, recent research has revealed that atonal syllables are not the minimum units that can induce behavioural facilitation in Chinese spoken word production^15, 16, 20–22^. For example, Wong and colleagues^16^ adopted the implicit priming paradigm to examine the effects of sharing word-initial body (i.e., onset + vowel; e.g. /**ja**p6 hau2/, /**ja**t1 cai3/, /**ja**n5 king4/, meaning “entrance”, “all”, and “engine”, respectively) and sharing atonal syllable on Cantonese (i.e., a Chinese dialect) spoken word production. Significant facilitation effects were found for both types of homogeneity, although sharing atonal syllables generated a larger effect than sharing word-initial bodies did. To account for these findings, at least two different hypotheses can be put forward. The first assumes that atonal syllables are the proximate phonological units in Chinese, and that the sub-syllabic effects may result from repeated segment retrieval in the segmental specification process following the selection of syllables. Hence, the mediation of syllabic processing makes sub-syllabic effects hard to be observed unless more than one segment is manipulated. Alternatively, segments instead of atonal syllables might be the proximate phonological units in Chinese, similar to that in Germanic languages. Due to the relatively simple syllable structure, Chinese speakers may retrieve and integrate segments in a highly efficient manner, resulting in the null onset effect. When the degree of segmental overlap is increased, its behavioural effects start to appear. These two hypotheses have not been verified. Therefore, it is still unclear what roles syllables and segments play in planning Chinese spoken word production.

On the other hand, in recent years some researchers have started to use the ERP technique to examine the effect of phonological overlap on real-time brain responses during overt spoken word production^23–27^. For example, in the study of Qu and colleagues^24^, participants were asked to produce colour-noun phrases upon picture prompts. They found that onset repetition between the colour name and the noun generated no behavioural differences but significantly modulated the ERPs in the 200- to 300-ms interval and the 300- to 400-ms interval after picture onset. In another picture naming task where each two consecutive pictures implicitly formed a pair of prime and target, onset repetition between the prime and the target induced similar ERP effects after target onset^26^. These researchers consequently proposed that the earlier ERP effect reflects a facilitatory effect of repeated segment retrieval and that the later ERP effect reflects increased workload in self-monitoring. Note that despite their claim that the functional engagement of segments in Chinese spoken word production does not contradict the assumption of syllables as the proximate phonological units in Chinese, the onset of segmental processing as early as 200 ms in their interpretation seems to support segments as the proximate phonological units. However, Chen and colleagues^12^ pointed out that syllable similarity resulting from segment repetition may influence syllabic processing as well and that the early ERP effect may alternatively reflect syllable selection instead of direct engagement with segments. Thus, it is important and useful to compare the ERP effect of syllable repetition with that of segment repetition for verifying the proximate phonological units in Chinese spoken word production.

In order to include a larger set of stimuli, the paradigm of primed picture naming^26^ was adopted in the current study to compare the ERP effects of syllabic and sub-syllabic repetition in Mandarin disyllabic word production for the first time. Participants were required to name pictures one by one, where each two consecutive pictures implicitly formed a pair of prime and target. Two types of phonological relatedness were designed for the picture pairs: 1) the two picture names shared the same first atonal syllable (e.g., /**jian**3dao1/, /**jian**4pan2/—“scissors”, “keyboard”), called the syllable-related condition; and 2) the first atonal syllable of one picture name consisted of the word-initial segments of the other picture name (e.g., /**xi**1gua1/, /**xi**n4feng1/—“watermelon”, “envelope”), called the body-related condition. Sub-syllabic overlap was maximized in the body-related condition so as to reduce the difference in the degree of segmental overlap between the two types of relatedness.

Additionally, a delayed naming procedure^28–30^ was adopted to avoid the problem of speech artifacts in the critical ERP waveforms, as well as to exclude any possible effect originating from motor execution. Although recent studies have started using the ERP technique to investigate the early processes in overt speech production^31–34^, these early brain responses are not necessarily free of artifacts. In an overt naming task with 850-ms average naming latency, Porcaro, Medaglia and Krott^35^ adopted an Independent Component Analysis procedure to remove articulation-related artifacts, and identified a major artifact after 400 ms post picture onset as well as a smaller but earlier artifact around 160 ms. To minimize the possible artifacts in the early processes, we chose to use a delayed naming task in the current study. Pictures stayed on the screen for 800 ms followed by a question mark (i.e., the cue), and participants were required to prepare the name of the picture as soon as possible but to withhold their naming response until the cue onset. Besides the main task, immediate picture naming was also required in filler trials^36^, in order to encourage immediate preparation of the naming response during the whole experiment. The same stimuli were used in the filler trials as in the experimental trials.

## Results

### Behavioural

The mean naming latency and error rate in the immediate naming task were 831 ms (SE = 20 ms) and 2.1% (SE = 0.3%) respectively. Specifically, the mean naming latency and error rate were 833 ms (SE = 20 ms) and 2.5% (SE = 0.5%) for the syllable-related picture set, and 828 ms (SE = 21 ms) and 1.7% (SE = 0.3%) for the body-related picture set. By-participants and by-items *t* tests showed no significant difference in naming latency (*p*s ≥ 0.338) or accuracy (*p*s ≥ 0.074), confirming that the participants responded similarly to these two sets of pictures.

In the delayed naming task, participants’ reaction time was measured from the onset of the response cue. The mean RTs for the target pictures were 406 ms (SE = 14 ms), 405 ms (SE = 14 ms), 408 ms (SE = 14 ms), and 408 ms (SE = 14 ms) in the syllable-related, syllable-unrelated, body-related, and body-unrelated conditions, while the error rates for the target pictures were 1.1% (SE = 0.3%), 0.9% (SE = 0.2%), 0.8% (SE = 0.2%), and 0.5% (SE = 0.2%) respectively. Two-way (prime-target relatedness × type of relatedness) ANOVAs (*F*_1_ by participants, *F*_2_ by items) demonstrated that no significant main effect or interaction was found either on the RTs (*F*_1_s ≤ 0.63, *p*s ≥ 0.434; *F*_2_s ≤ 0.43, *p*s ≥ 0.513) or on the error rates (*F*_1_s ≤ 3.04, *p*s ≥ 0.092; *F*_2_s ≤ 2.74, *p*s ≥ 0.102).

### ERPs

**Figure 1:**
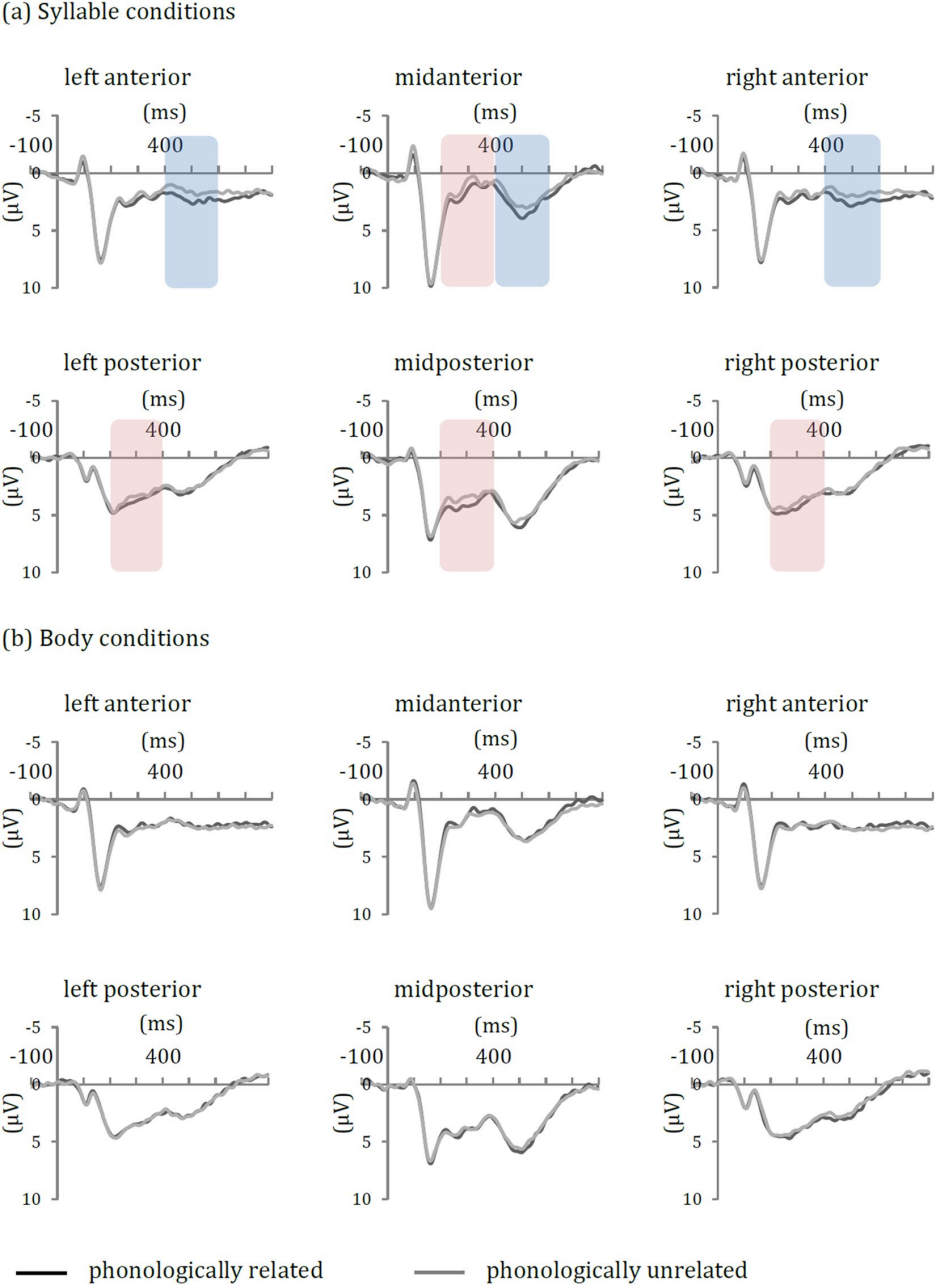
Grand average ERPs in (**a**) the syllable conditions and (**b**) the body conditions at six ROIs: left anterior (F5, F7, FC5), midanterior (Fz, FCz, Cz), right anterior (F6, F8, FC6), left posterior (P5, P7, CP5), midposterior (CPz, Pz, POz) and right posterior (P6, P8, CP6) regions. Only syllable-relatedness induced significant ERP effects after target onset: a widely distributed positivity in the 200- to 400-ms interval (red shading) and an anterior positivity in the 400- to 600-ms interval (blue shading).

Figure 1 displays the grand average ERPs at each ROI. Four-way (prime-target relatedness × type of relatedness × anteriority × laterality) repeated-measures ANOVAs were performed on the mean amplitude in each consecutive 200-ms time interval after picture onset, as summarized in Table 1. No main effect or interaction involving prime-target relatedness was significant in the time window of 0–200 ms (*F*s ≤ 1.32, *p*s ≥ 0.272).

**Table 1:** Summary of omnibus ANOVA and post hoc *t* test results on mean ERP amplitude. Ant: anteriority; Lat: laterality; Rel: prime-target relatedness; Typ: type of relatedness; —: non-significant *F* or *t* value, *p* ≥ 0.1. †*p* < 0.1, *p < 0.05, **p < 0.01.

	0–200 ms	200–400 ms	400–600 ms	600–800 ms
Omnibus ANOVA				
Rel (1, 29)	—	—	—	—
Rel*Typ (1, 29)	—	4.30*	—	—
Rel*Ant (1, 29)	—	—	—	—
Rel*Lat (2, 58)	—	—	—	—
Rel*Typ*Ant (1, 29)	—	—	6.67*	3.16†
Rel*Typ*Lat (2, 58)	—	—	—	—
Rel*Ant*Lat (2, 58)	—	—	2.45†	2.99†
Rel*Typ*Ant*Lat (2, 58)	—	—	—	—
Effect of syllable-relatedness at individual ROIs				
Left anterior (1, 29)	—	—	2.79**	—
Midline anterior (1, 29)	—	2.16*	1.84†	—
Right anterior (1, 29)	—	—	2.37*	—
Left posterior (1, 29)	—	2.37*	—	—
Midline posterior (1, 29)	—	3.09**	—	—
Right posterior (1, 29)	—	2.30*	—	—
Effect of body-relatedness at individual ROIs				
Left anterior (1, 29)	—	—	—	—
Midline anterior (1, 29)	—	—	—	—
Right anterior (1, 29)	—	—	—	—
Left posterior (1, 29)	—	—	—	—
Midline posterior (1, 29)	—	—	—	—
Right posterior (1, 29)	—	—	—	—

In the 200- to 400-ms time window, the interaction of prime-target relatedness and type of relatedness was significant (*F*_(1,29)_ = 4.30, *p* = 0.047), and the main effect of prime-target relatedness and other interactions involving prime-target relatedness were not significant (*F*s ≤ 1.50, *p*s ≥ 0.231). Follow-up pairwise comparisons showed that the ERP waveform was significantly more positive in the syllable-related condition relative to the syllable-unrelated condition (*p* = 0.003), but not significantly different between the body-related and the body-unrelated conditions (*p* = 0.769). Planned pairwise comparisons at each ROI demonstrated that the syllable effect was significant in the midanterior (*p* = 0.039), left posterior (*p* = 0.025), midposterior (*p* = 0.004), and right posterior (*p* = 0.029) regions, and that no significant effect of body-relatedness was found in any ROI (*p*s ≥ 0.404). In addition, a significant effect of syllable-relatedness was also found when a larger set of 50 electrodes (FZ, F1, F2, F3, F4, F5, F6, F7, F8, FCZ, FC1, FC2, FC3, FC4, FC5, FC6, FT7, FT8, CZ, C1, C2, C3, C4, C5, C6, T7, T8, CPZ, CP1, CP2, CP3, CP4, CP5, CP6, TP7, TP8, PZ, P1, P2, P3, P4, P5, P6, P7, P8, POZ, PO3, PO4, PO7, PO8) were analyzed (relatedness: *F*_(1,29)_ = 11.70, *p* = 0.002; relatedness × electrode: *F*_(49,1421)_ = 0.85, *p* = 0.494), but not for body-relatedness (relatedness: *F*_(1,29)_ = 0.13, *p* = 0.721; relatedness × electrode: *F*_(49,1421)_ = 1.48, *p* = 0.213).

In the 400- to 600-ms time window, the three-way interaction of prime-target relatedness, type of relatedness and anteriority was significant (*F*_(1,29)_ = 6.67, *p* = 0.015), and the three-way interaction of prime-target relatedness, anteriority and laterality was marginally significant (*F*_(2,58)_ = 2.45, *p* = 0.095). The main effect of prime-target relatedness and other interactions involving prime-target relatedness were not significant (*F*s ≤ 2.18, *p*s ≥ 0.151). Follow-up pairwise comparisons revealed a significant positive effect of syllable-relatedness in the anterior regions (*p* = 0.013), but not in the posterior regions (*p* = 0.389). In contrast, no significant effect was found for body-relatedness (anterior regions: *p* = 0.744; posterior regions: *p* = 0.480). Planned pairwise comparisons at each ROI further demonstrated that only syllable-relatedness had a significant (or marginally significant) effect, in the left anterior (*p* = 0.009), midanterior (*p* = 0.076), and right anterior (*p* = 0.025) regions (body-relatedness: *p*s ≥ 0.212). Similar results were obtained when a larger set of anterior electrodes (FZ, F1, F2, F3, F4, F5, F6, F7, F8, FCZ, FC1, FC2, FC3, FC4, FC5, FC6, FT7, FT8, CZ, C1, C2, C3, C4, C5, C6, T7, T8) were included in analyses (syllable-relatedness: *F*_(1,29)_ = 6.15, *p* = 0.019; body-relatedness: *F*_(1,29)_ = 0.08, *p* = 0.777).

In the 600- to 800-ms time window, the three-way interaction of prime-target relatedness, type of relatedness and anteriority (*F*_(1,29)_ = 3.16, *p* = 0.086), as well as the three-way interaction of prime-target relatedness, anteriority and laterality (*F*_(2,58)_ = 2.99, *p* = 0.058), was marginally significant. However, no significant syllable effect or body effect was found in follow-up pairwise comparisons (anterior regions: *p*s ≥ 0.304; posterior regions: *p*s ≥ 0.910; each ROI: *p*s ≥ 0.203).

## Discussion

The current study compared for the first time the ERP effects of repeating atonal syllables versus sub-syllabic components in Mandarin spoken word production. Participants produced disyllabic words sequentially upon picture prompts while each two consecutive pictures implicitly formed a pair of prime and target. Note that since no written or spoken materials were included in the entire procedure, production-specific processes were not contaminated by word or speech perception. Significant ERP effects of syllable-relatedness between the paired pictures were observed in two time intervals after target onset: A widely distributed positivity in the 200- to 400-ms interval and an anterior positivity in the 400- to 600-ms interval. In contrast, no significant effects were found for body-relatedness in any time interval. The implications of these results are discussed below.

First of all, the current study adopted a delayed naming procedure, in which participants were required to withhold their naming responses until the onset of a response cue (i.e., 800 ms after picture onset). The mean latency of naming the same set of pictures without delay was 831 ms, as shown in the filler trials. Since the execution of naming responses took more than 31 ms^30, 37, 38^, the 800-ms delay must have been adequate for the pre-articulatory processes. Besides, participants’ reaction time to the cue was very short (i.e., around 400 ms) and was not influenced by the relatedness between the prime and the target, indicating that the naming responses were well prepared and initiated once the cue was detected. Thus, it is believed that all the cognitive processes involved in picture naming, except the execution of motor programs, were completed before the onset of the cue in a similar way as in immediate picture naming.

With the above assumption, the ERP effect of syllable-relatedness in the 200- to 400-ms interval is localized at the beginning of the phonological encoding stage. Based on a comprehensive meta-analysis of neurocognitive studies on word production, Indefrey and Levelt^37, 38^ estimated that for an average disyllabic word with 5 segments phonological encoding may take place 275–455 ms after picture onset, largely overlapped with the current interval of 200–400 ms. The wide distribution of this positivity is also consistent with previous findings about phonological effects on ERP during overt spoken word production^23, 25–27^. The null effect of body-relatedness in this time window is against the prediction of segments as the proximate phonological units for Mandarin Chinese. Our design has maximized the sub-syllabic relatedness in the body-related condition, and previous studies have revealed that this degree of segmental overlap is sufficient to generate a reliable facilitation in performance^15, 16, 20–22^. If the shared segments are repeatedly retrieved as the proximate phonological units, it is unlikely to observe a null effect for body-relatedness while a widely distributed ERP effect was found for syllable-relatedness. Thus, this positivity should have resulted from repeated retrieval of atonal syllables at the beginning of phonological encoding, supporting atonal syllables as the proximate phonological units in Mandarin spoken word production.

The anterior positivity in the later time interval was also induced by syllable-relatedness only. This might result from the phonetic encoding stage, where articulatory gestures are selected for the output of phonological encoding. This explanation is consistent with an influential model WEAVER++^39^ which assumes that the gestural scores for frequent syllables are stored in a syllabary^40–42^ and accessed during phonetic encoding. Hence, the anterior positivity may reflect repeated retrieval of syllabic gestures so that body-relatedness did not produce a significant effect. Another possible account concerns the retention of retrieved syllables during the delay period. The occurrence of the above phonological effect as early as 200 ms after picture onset suggested that in spite of the delayed naming requirement the participants started the phonological encoding process once possible. The output of phonological encoding might be maintained in working memory before being further processed. According to the working memory model of Baddeley and Hitch^43, 44^, phonological information can be maintained in a phonological store for a few seconds and be refreshed via articulatory rehearsal. The anterior positivity may be induced by the maintenance of a syllable-sized representation sharing the first atonal syllable with the one in the preceding trial, relative to an unrelated control. Note that these two accounts are not mutually exclusive. Rather than maintaining the phonological representation, the participants may continue to prepare the phonetic representation and then maintain it via articulatory rehearsal in working memory.

Our finding that body-relatedness induced no ERP effect seems to be inconsistent with the previous findings^24, 26^. The absence of a reliable sub-syllabic effect in the present study does not necessarily mean that segments have no function in planning Mandarin spoken word production. One possible account is that due to the delayed naming requirement the time interval between prime onset and target onset was lengthened relative to the original procedure^26^. The priming effect of segmental repetition might have been reduced to the baseline level, showing no observable effect, but interestingly, the effects of syllabic repetition were not affected. Future studies may consider replicating the current study with a covert naming task instead of the delayed naming task so as to shorten the interval between the prime and the target.

Nevertheless, caution is warranted in interpreting the ERP effects of segment repetition in the previous studies^24, 26^. The current results clearly suggest that atonal syllables are selected as the proximate phonological units since 200 ms after picture onset. Thus, the first time window of their ERP effects (i.e., 200–300 ms) may be too early for segmental processing to take place. It is more likely to reflect facilitation or competition between similar syllable nodes in the syllabic processing stage^12^. Since segmental specification is mediated by syllable selection, an ERP effect induced by repeated segment retrieval, if any, might fall in the second time window of their ERP effects (i.e., 300–400 ms), which was interpreted as increased workload in self-monitoring in previous work^24, 26^. Self-monitoring refers to speakers’ ability to perceive their inner or overt speech for self-repair^45^. Qu and colleagues^24^ proposed that segment repetition might increase the workload of the monitoring system and thus cause an ERP modulation. To tell apart the segment retrieval account from the self-monitoring account, techniques with finer spatial resolution may provide additional information, such as fMRI^46^ and TMS^47^.

The current results together with previous findings indicate that the proximate phonological units in spoken word production are language-specific (i.e., atonal syllables in Mandarin Chinese and segments in Germanic languages). This difference might be due to the following reasons. First, resyllabification is very common in Germanic languages. For instance, the segment /t/ is a syllable coda in the English word *get*, but becomes onset of the next syllable in the phrase *get it* (“ge-tit”). Using segments as the proximate units is more flexible and meets the need of resyllabification. In contrast, there is no such need in Chinese word production. Chinese syllables have clear boundaries, and each logogram in the Chinese orthography corresponds to a separate syllable. In addition, the number of legitimate syllables in Mandarin Chinese is very limited as compared with that in Germanic languages. Thus, it may be relatively easier and more efficient to store atonal syllables as the proximate units in Mandarin.

To conclude, the current study provides the first electrophysiological evidence for the distinct role of syllables at the phonological encoding stage of producing Mandarin spoken words. It supplies support for the language specificity hypothesis about the proximate phonological units in spoken word production^9^.

## Methods

### Participants

Thirty-six native Mandarin speakers (14 males; mean age = 20 years, SD = 0.9 year) were recruited from South China Normal University. They had normal or corrected-to-normal vision, without a history of any psychiatric or neurological disorder. Informed consent was obtained from each participant at the beginning of the experiment, and their participation was rewarded by 80 RMB (i.e., around 12 USD). This study adheres to the ethical procedures for the protection of human participants in research and was approved by the ethics committee of the School of Psychology at South China Normal University.

### Stimuli

Thirty-six pairs of white-on-black line drawings of common objects were used as stimuli, and each picture was assigned with a disyllabic Mandarin name (see Supplementary Table S1). For half of the pairs (i.e., 18 pairs), the two pictures within a pair shared the first atonal syllable of their names (e.g., /**jian**3dao1/, /**jian**4pan2/—scissors, keyboard), constituting the syllable-related condition. For the other half, the first atonal syllable of one picture name consisted of the word-initial segments of the other picture name (e.g., /**xi**1gua1/, /**xi**n4feng1/—watermelon, envelope), constituting the body-related condition. These two sets of pictures were matched in their name frequency and first syllable frequency irrespective of tones^48^, *t*s ≤ 1.10, *p*s ≥ 0.274. Pictures in each set were also recombined with each other to generate the corresponding unrelated conditions (i.e., syllable-unrelated, body-unrelated). In any condition, the picture names within a pair were not related orthographically. Besides, sixteen native Mandarin speakers who did not participate in the main experiment were asked to rate on semantic relatedness between each pair of pictures with a 5-point scale. While 1 indicated that the two pictures were not semantically related at all, the average score of picture pairs in the syllable-related, syllable-unrelated, body-related and body-unrelated conditions were 1.3 (SD = 0.3), 1.4 (SD = 0.3), 1.5 (SD = 0.3) and 1.4 (SD = 0.3), respectively. So the semantic relatedness of picture pairs was controlled at a considerably low level and comparable across all the conditions. For each pair of pictures, both ways of ordering (i.e., A primes B, B primes A) were adopted so that all pictures acted as primes as well as targets.

### Design and procedure

The experiment adopted a two-factor within-participants design (prime-target relatedness: phonologically related vs. unrelated; type of relatedness: syllable, body). All picture pairs appeared in both ways of ordering with one repetition, resulting in 72 trials (18 pairs * 2 orderings * 2 times) for each condition. Each of the 72 pictures also acted as fillers twice. Twenty-four blocks were generated so that each block contains three experimental trials from each condition and six filler trials in a randomized order. The order of blocks was also randomized for each participant.

**Figure 2:**
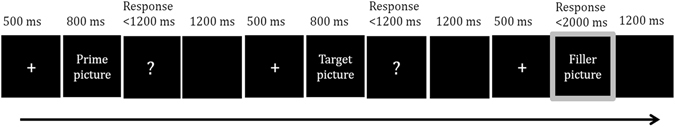
Procedure of the experimental trial and the filler trial. In the experimental trials, the prime picture and the target picture were presented alone sequentially. In the filler trials, the picture was presented together with a gray frame. Participants were required to withhold their naming responses until the onset of a question mark when the picture was presented alone, and to name the picture immediately when the picture was accompanied with a gray frame.

In each experimental trial, the prime picture and the target picture were presented and named sequentially (Fig. 2). After a 500-ms white fixation, the picture (expanding approximately 5° × 5° in visual angle) was presented alone and stayed at the center of the screen for 800 ms. The participants were required to prepare the name of the picture as quickly as possible, but to withhold their naming responses until the onset of a question mark. The question mark stayed on the screen for 1200 ms or until a naming response was detected by the voicekey, followed by a 1200-ms blank. Participants’ reaction time was measured from the onset of the question mark. In the filler trials, the picture appeared together with a gray frame and stayed on the screen for 2000 ms or until a naming response was produced. Participants were asked to name the picture as accurately and quickly as possible when the picture was accompanied with a gray frame, and their naming latencies were measured from the onset of the picture. Before the test phase, participants first learned the names of the 72 pictures and did a practice session (including both immediate naming and delayed naming trials) upon successful learning. The experiment lasted for approximately 60 minutes, with short breaks between blocks.

### EEG recording and pre-processing

Electroencephalogram (EEG) from 64 scalp sites (according to the 10–10 system convention, FCz as online reference) and electrooculogram (EOG) signals were recorded during the experiment, with a sampling rate of 500 Hz. The impedance of each electrode was kept below 5 kΩ. Offline analyses of EEG signals were carried out with the EEGLAB toolbox^49^. The EEG signals were re-referenced to the average of the left and right mastoids, and bandpass (0.1–30 Hz) filtered. Eye movement artifacts were corrected using the EMCP algorithm^50^. Then 900-ms epochs were obtained relative to the onset of target pictures, including 100-ms pre-stimulus baselines. After baseline correction, epochs containing amplifier blocking, artifacts exceeding ±80 μV, or electrode drifting were rejected before ERP calculation. Data of six participants (two males) were discarded due to bad data quality. Besides, incorrect responses (0.8%), responses earlier than the cue onset (4.1%) or exceeding 2.5 SD of the individual’s mean reaction time (4.5%) were excluded in both behavioural and ERP analyses. The remained epochs were averaged to obtain ERPs for each participant. As in previous studies^24, 26^, ERP waveforms at six regions of interest (ROIs) were computed by further averaging ERPs from proximal electrodes: left anterior (F5, F7, FC5), midanterior (Fz, FCz, Cz), right anterior (F6, F8, FC6), left posterior (P5, P7, CP5), midposterior (CPz, Pz, POz) and right posterior (P6, P8, CP6) regions.

### Data availability

The data that support the findings of the current study are available from the first author upon reasonable request.

## Electronic supplementary material

Supplementary information
