# Supplementary material for: Primary phonological planning units in spoken word production are language-specific: Evidence from an ERP study
Source: Sci Rep. 2017 Jul 19;7:5815. doi: 10.1038/s41598-017-06186-z (PMC5517664; doi:10.1038/s41598-017-06186-z)
Supplement: Supplementary file 1 — Supplementary information [file 41598_2017_6186_MOESM1_ESM.pdf]

Supplementary Material for *Scientific Reports*

Primary phonological planning units in spoken word production are language-specific: Evidence from an ERP study

Jie Wang[1], Andus Wing-Kuen Wong[2], Suiping Wang[3], and Hsuan-Chih Chen[1,*]

[1]Department of Psychology, The Chinese University of Hong Kong, Hong Kong S.A.R., China

[2]Nam Shan Psychology Laboratory, Department of Applied Social Sciences, City University of Hong Kong, Hong Kong S.A.R., China

[3]Department of Psychology, South China Normal University, Guangzhou 510631, China

[*]hcchen@psy.cuhk.edu.hk

Related Pairs		Unrelated Pairs	
Syllable-relatedness			
刺猬 /ci4wei4/<br>*hedgehog*	磁带 /ci2dai4/<br>*tape*	刺猬 /ci4wei4/<br>*hedgehog*	胶囊 /jiao1nang2/<br>*capsule*
灯泡 /deng1pao4/<br>*light bulb*	凳子 /deng4zi/<br>*stool*	灯泡 /deng1pao4/<br>*light bulb*	兔子 /tu4zi/<br>*rabbit*
拱桥 /gong3qiao2/<br>*bridge*	公鸡 /gong1ji1/<br>*cock*	拱桥 /gong3qiao2/<br>*bridge*	扇子 /shan4zi/<br>*fan*
滑梯 /hua2ti1/<br>*slide*	花瓶 /hua1ping2/<br>*vase*	滑梯 /hua2ti1/<br>*slide*	键盘 /jian4pan2/<br>*keyboard*
剪刀 /jian3dao1/<br>*scissors*	键盘 /jian4pan2/<br>*keyboard*	剪刀 /jian3dao1/<br>*scissors*	咖啡 /ka1fei1/<br>*coffee*
教室 /jiao4shi4/<br>*classroom*	胶囊 /jiao1nang2/<br>*capsule*	教室 /jiao4shi4/<br>*classroom*	蘑菇 /mo2gu1/<br>*mushroom*
卡车 /ka3che1/<br>*truck*	咖啡 /ka1fei1/<br>*coffee*	卡车 /ka3che1/<br>*truck*	寿司 /shou4si1/<br>*sushi*
孔雀 /kong3que4/<br>*peacock*	空调 /kong1tiao2/<br>*air conditioner*	孔雀 /kong3que4/<br>*peacock*	硬币 /ying4bi4/<br>*coin*
蜜蜂 /mi4feng1/<br>*bee*	迷宫 /mi2gong1/<br>*maze*	蜜蜂 /mi4feng1/<br>*bee*	烟囱 /yan1cong1/<br>*chimney*
墨水 /mo4shui3/<br>*ink*	蘑菇 /mo2gu1/<br>*mushroom*	墨水 /mo4shui3/<br>*ink*	指纹 /zhi3wen2/<br>*fingerprint*
闪电 /shan3dian4/<br>*lightning*	扇子 /shan4zi/<br>*fan*	闪电 /shan3dian4/<br>*lightning*	空调 /kong1tiao2/<br>*air conditioner*
手枪 /shou3qiang1/<br>*pistol*	寿司 /shou4si1/<br>*sushi*	手枪 /shou3qiang1/<br>*pistol*	花瓶 /hua1ping2/<br>*vase*
土豆 /tu3dou4/<br>*tomato*	兔子 /tu4zi/<br>*rabbit*	土豆 /tu3dou4/<br>*tomato*	公鸡 /gong1ji1/<br>*cock*
拖鞋 /tuo1xie2/<br>*slipper*	鸵鸟 /tuo2niao3/<br>*ostrich*	拖鞋 /tuo1xie2/<br>*slipper*	凳子 /deng4zi/<br>*stool*
香蕉 /xiang1jiao1/<br>*banana*	橡皮 /xiang4pi2/<br>*eraser*	香蕉 /xiang1jiao1/<br>*banana*	磁带 /ci2dai4/<br>*tape*
眼镜 /yan3jing4/<br>*glasses*	烟囱 /yan1cong1/<br>*chimney*	眼镜 /yan3jing4/<br>*glasses*	鸵鸟 /tuo2niao3/<br>*ostrich*
鹦鹉 /ying1wu3/<br>*parrot*	硬币 /ying4bi4/<br>*coin*	鹦鹉 /ying1wu3/<br>*parrot*	迷宫 /mi2gong1/<br>*maze*
知了 /zhi1liao3/<br>*cicada*	指纹 /zhi3wen2/<br>*fingerprint*	知了 /zhi1liao3/<br>*cicada*	橡皮 /xiang4pi2/<br>*eraser*
Body-relatedness			
鼻子 /bi2zi/<br>*nose*	冰箱 /bing1xiang1/<br>*refrigerator*	鼻子 /bi2zi/<br>*nose*	碗筷 /wan3kuai4/<br>*dishes*
企鹅 /qi3e2/<br>*penguin*	铅笔 /qian1bi3/<br>*pencil*	企鹅 /qi3e2/<br>*penguin*	帽子 /mao4zi/<br>*hat*

牙膏 /ya2gao1/	钥匙 /yao4shi/	牙膏 /ya2gao1/	窗帘 /chuang1lian2/
toothpaste	*key*	*toothpaste*	*curtain*
厨师 /chu2shi1/	窗帘 /chuang1lian2/	厨师 /chu2shi1/	信封 /xin4feng1/
cook	*curtain*	*cook*	*envelope*
蝴蝶 /hu2die2/	火柴 /huo3chai2/	蝴蝶 /hu2die2/	睡衣 /shui4yi1/
butterfly	*matches*	*butterfly*	*pajamas*
礼物 /li3wu4/	流星 /liu2xing1/	礼物 /li3wu4/	圆规 /yuan2gui1/
gift	*meteor*	*gift*	*divider*
鸡蛋 /ji1dan4/	奖杯 /jiang3bei1/	鸡蛋 /ji1dan4/	钥匙 /yao4shi/
egg	*trophy*	*egg*	*key*
蚂蚁 /ma3yi3/	帽子 /mao4zi/	蚂蚁 /ma3yi3/	流星 /liu2xing1/
ant	*hat*	*ant*	*meteor*
袜子 /wa4zi/	碗筷 /wan3kuai4/	袜子 /wa4zi/	苹果 /ping2guo3/
sock	*dishes*	*sock*	*apple*
书包 /shu1bao1/	睡衣 /shui4yi1/	书包 /shu1bao1/	铁链 /tie3lian4/
schoolbag	*pajamas*	*schoolbag*	*chain*
西瓜 /xi1gua1/	信封 /xin4feng1/	西瓜 /xi1gua1/	火柴 /huo3chai2/
watermelon	*envelope*	*watermelon*	*matches*
羽毛 /yu3mao2/	圆规 /yuan2gui1/	羽毛 /yu3mao2/	冰箱 /bing1xiang1/
feather	*divider*	*feather*	*refrigerator*
大象 /da4xiang4/	岛屿 /dao3yu3/	大象 /da4xiang4/	铅笔 /qian1bi3/
elephant	*island*	*elephant*	*pencil*
披萨 /pi1sa4/	苹果 /ping2guo3/	披萨 /pi1sa4/	老鼠 /lao3shu3/
pizza	*apple*	*pizza*	*mouse*
吸管 /xi1guan3/	小丑 /xiao3chou3/	吸管 /xi1guan3/	岛屿 /dao3yu3/
straw	*clown*	*straw*	*island*
辣椒 /la4jiao1/	老鼠 /lao3shu3/	辣椒 /la4jiao1/	章鱼 /zhang1yu2/
pepper	*mouse*	*pepper*	*octopus*
梯子 /ti1zi/	铁链 /tie3lian4/	梯子 /ti1zi/	奖杯 /jiang3bei1/
ladder	*chain*	*ladder*	*trophy*
炸弹 /zha4dan4/	章鱼 /zhang1yu2/	炸弹 /zha4dan4/	小丑 /xiao3chou3/
bomb	*octopus*	*bomb*	*clown*

Supplementary Table S1. Picture pairs used in the four conditions (picture names in Simplified Chinese, English translations in italics, Pinyin representations between slashes; the numbers denote the type of tones). Pictures in the related conditions were recombined with each other to generate the corresponding unrelated conditions.
